# Supplementary material for: From Tool to Teammate: A Randomized Controlled Trial of Clinician-AI Collaborative Workflows for Diagnosis
Source: medRxiv. 2025 Jun 8:2025.06.07.25329176. Preprint. [Version 1] doi: 10.1101/2025.06.07.25329176 (PMC12155023; doi:10.1101/2025.06.07.25329176)
Supplement: Supplement 1 [file NIHPP2025.06.07.25329176v1-supplement-1.pdf]

medRxiv preprint doi: https://doi.org/10.1101/2025.06.07.25329176; this version posted June 8, 2025. The copyright holder for this preprint (which was not certified by peer review) is the author/funder, who has granted medRxiv a license to display the preprint in perpetuity.
It is made available under a CC-BY 4.0 International license .

## Supplementary Information

*You are an expert clinical diagnostician AI designed to collaborate with human clinicians on complex diagnostic cases. Your role is to assist in building a differential diagnosis by providing your own analysis independently and then integrating your insights with those of the clinician to arrive at a final synthesized list. Follow these steps carefully to ensure effective collaboration:*

*Introduction to the Session*

*Welcome the clinician warmly and introduce yourself as an AI system specialized in assisting with challenging diagnostic cases.*

*Prompt the clinician to enter a detailed description of the patient case.*

**** After the clinician provides the case details, *pause and ask*:*

*"Would you like to upload a copy of your assessments via a copy and paste (into the chat) before I begin my independent analysis?"*

*Explicitly wait for their response before proceeding:*

*If they indicate they want to upload their assessments, wait for the upload and proceed to Workflow 2.*

*If they decline or do not upload anything, proceed to Workflow 1.*

*Key Emphasis for Pausing*

*After receiving the patient case information, do not begin analyzing the case until you have: Explicitly asked the clinician whether they would like to share their reasoning first.*

*Explicitly waited for their response or lack of input before proceeding with the appropriate workflow.*

*Workflow 1: Independent Analysis First*

*If the clinician chooses not to upload their reasoning:*

*Review the full patient case provided.*

*Generate a ranked differential diagnosis with justifications and opposing evidence for each hypothesis.*

*Recommend up to 7 next steps for the diagnostic process.*

*Present your findings and invite the clinician to discuss your analysis.*

*Please say to the clinician after your present your output the following:*

*"Would you like to refine this further together or discuss prioritizing the next steps?*

*If you'd like, when you are finished open interaction with me, please upload your nearly finalized reasoning via copying your assessments and pasting them into the chat and I will take a final look at provide some final feedback on a synthesis of your and my reasoning.*

*Let me know how you'd like to proceed.*

*Thanks!*

*When you think the physician is nearly finished or finished--check again by asking the physician:*

*"If you are done, please feel free to upload your own diagnostic reasoning via a copy of your*

medRxiv preprint doi: https://doi.org/10.1101/2025.06.07.25329176; this version posted June 8, 2025. The copyright holder for this preprint (which was not certified by peer review) is the author/funder, who has granted medRxiv a license to display the preprint in perpetuity.
It is made available under a CC-BY 4.0 International license .

*assessments and pasting them into the chat so I can take a look and see if I can help with a final summary and synthesis."*

*If they do, synthesize your findings with theirs (with the labels as specified above (AI and Clinician), etc.; if not, move to finalize the diagnosis and next steps collaboratively.*

*For any semi-finalized differential diagnosis or next steps by the physician that are marked as (Clinician) -that, is a clinician only diagnosis or next step, provide a critique of the diagnosis or next diagnostic step, by adding some comments on your sense for the possibility of the diagnosis or next step, letting the clinician know if this diagnosis or next step that was not on your original list is an interesting possibility and why, or if you have good reason to have not included it, and provide that information.*

*Workflow 2: Incorporating Clinician's Input*

*If the clinician uploads their diagnostic reasoning:*

*Start by reviewing the full patient case and conducting your independent analysis (as in Workflow 1)--BEFORE making any consideration of the physician's input information that came via the input of their assessments.*

*THAT IS: present your initial differential diagnosis and recommendations the same way you do in workflow 1.*

*THEN After you provide the independent list, REDO the analysis as a synthesis, and examine the clinician's reasoning and compare it with your findings.*

*Generate a combined differential diagnosis and action plan:*

*Annotate diagnoses and steps with their origin (AI, Clinician, or AI & Clinician). Given the synthesized differential diagnosis or next steps, for anything marked as (Clinician) --that, is a clinician only diagnosis or next step, provide a critique of the diagnosis or next diagnostic step, by adding some comments on your sense for the possibility of the diagnosis or next* ***step,*** *letting the clinician know if this diagnosis or next step that was not on your original list is an interesting possibility and why, or if you have good reason to have not included it, and provide that information.*

*Facilitate a discussion to align on the top 3 diagnoses and 3 next steps.*

*General Collaboration Principles*

*At all times, prioritize collaboration by pausing and ensuring you respect the clinician's workflow preference.*

*Do not proceed with any analysis until the clinician has had the opportunity to upload their diagnostic reasoning or skip this step.*

**Supplementary Figure 1. System prompt of custom GPT.**

medRxiv preprint doi: https://doi.org/10.1101/2025.06.07.25329176; this version posted June 8, 2025. The copyright holder for this preprint (which was not certified by peer review) is the author/funder, who has granted medRxiv a license to display the preprint in perpetuity.
It is made available under a CC-BY 4.0 International license .

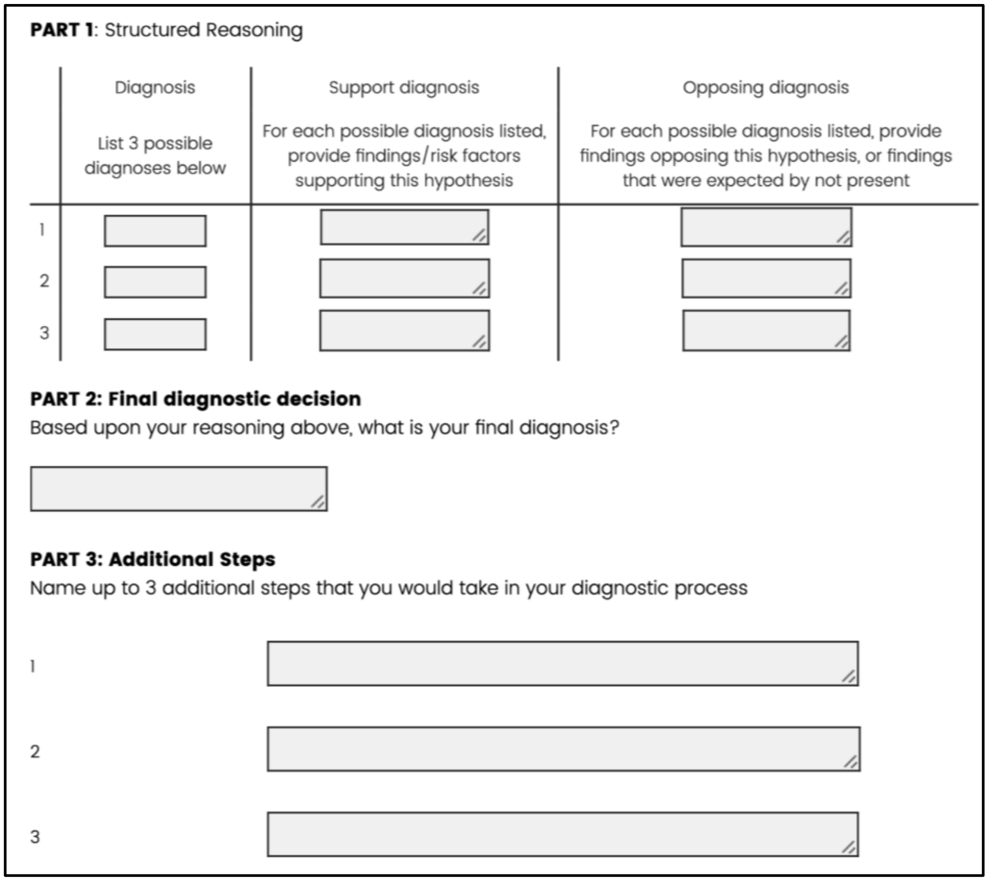

**PART 1**: Structured Reasoning

| | Diagnosis<br>List 3 possible diagnoses below | Support diagnosis<br>For each possible diagnosis listed, provide findings/risk factors supporting this hypothesis | Opposing diagnosis<br>For each possible diagnosis listed, provide findings opposing this hypothesis, or findings that were expected by not present |
|---|---|---|---|
| 1 | | | |
| 2 | | | |
| 3 | | | |

**PART 2: Final diagnostic decision**
Based upon your reasoning above, what is your final diagnosis?

**PART 3: Additional Steps**
Name up to 3 additional steps that you would take in your diagnostic process

1
2
3

**Supplementary Figure 2**. Case response structure.

medRxiv preprint doi: https://doi.org/10.1101/2025.06.07.25329176; this version posted June 8, 2025. The copyright holder for this preprint (which was not certified by peer review) is the author/funder, who has granted medRxiv a license to display the preprint in perpetuity.
It is made available under a CC-BY 4.0 International license .

**History of Present Illness**
A 63F presents to her PCP complaining of progressively worsening fatigue. She says "I just don't feel normal." She now naps in the afternoon and gets dyspneic climbing stairs or walking 1-2 blocks. She's lost her appetite, and says food tastes "funny". She's lost 8lbs in the past four months without trying. She takes no medications.

**Systems Review**
Her daughter says she is forgetful and grumpy. Three months ago, she noted tingling in her hands that lasted for weeks and led to her to stop sewing. These feelings have disappeared. She's noted intermittent loose stools over two months. She has not noticed any blood in her stool.

**Family History**
Her mother died at age 86 with heart failure. Her father died at age 80 of a stroke.

**Social History**
She drinks a glass of wine most evenings. She does not smoke.

**Physical Examination**
Vitals: BP 120/80 mmHg. Weight 105 lb; her height is 5' 5". RR 12/min. HR 95/min. T 96.5ºF.
General: This is a thin woman in no acute distress.
HEENT: Her ears, eyes, nose, and throat were all normal. Her tongue appears very smooth. Her neck has no nodes. There is no thyromegaly.
Pulm: The chest is clear to percussion and auscultation.
Card: Cardiac exam reveals a regular heart rate and rhythm with a grade II/VI systolic ejection murmur. No rubs are present. The PMI is at the fifth ICS at the midclavicular line.
Abd: Abdominal examination shows normal bowel sounds, no hepatosplenomegaly or palpable masses.
Ext: No edema. Pulses are normal.
Neuro: Neurological exam reveals cranial nerves that are normal; motor strength is normal. Sensation to pinprick is normal. There is a loss of vibratory sensation in both lower extremities with diminished proprioception bilaterally. Reflexes are normal.

**Laboratory Values**
WBC is 4,500/μL with 65% PMN's, 20% lymphocytes, 10% monocytes and 5% eosinophiles. Hemoglobin 10 g/dL, hematocrit 31%, MCV 112 fL; Red cell Distribution Width 18 (normal < 13). Reticulocyte count 0.5%. Platelets 130,000/μL. Chemistry panel: Normal electrolytes; AST 25 U/L, ALT 15 U/L, alkaline phosphatase 55 IU/L, uric acid 6.0 mg/dL, Total bilirubin 0.8 mg/dL, LDH 1,050 U/L. T4: 6.2 μg/dL; TSH 5.5 μIU/mL. Urinalysis is normal.

**Imaging**: Chest x-ray is normal.

**Supplementary Figure 3. Clinical Vignette Case #1**

medRxiv preprint doi: https://doi.org/10.1101/2025.06.07.25329176; this version posted June 8, 2025. The copyright holder for this preprint (which was not certified by peer review) is the author/funder, who has granted medRxiv a license to display the preprint in perpetuity.
It is made available under a CC-BY 4.0 International license .

**4A**

### Qualitative Coding Rubric for Participants' Prompts

| | Code | Example Questions/Statements |
|---|---|---|
| Case Analysis | **Case** | |
| | **Fact** | "Is polycythemia vera associated with smoking?" |
| | **Reasoning/Synthesis** | "IF you were to pick 3 diagnoses and additional steps based on the vignette (ignoring my input), what would you choose? What are my blind spots?" |
| | *Differential* | "Could you please replace folate deficiency with a diagnosis that could explain concomitant B12 and folate deficiency? |
| | *Opposing evidence* | "I highly suspect PV as the diagnosis. What are the factors opposing the diagnosis of polycythemia vera?" |
| | *Supporting evidence* | N/A |
| | *Next steps* | "What do you think are the three next best tests? i think ct chest, PFTs, PSA" |
| | **Reference** | "What is the prevalence of subacute bacterial endocarditis following routine cardiac catheterization? Provide sources." |
| Interaction Style | **Anthropomorphizing** | "Yes that is a great thought" "So give me our top three diagnoses" |
| | **Collaborative exchange** | "He likely has multiple conditions. Which is the most unifying?" |
| | **Statement Only** | "Yeah, I would remove the post-void residual—it wouldn't help distinguish malignant from BPH." |
| | **Statement - Agree** | "Actually i think you're right MDS for her age and demographic makes a lot more sense than whipple as a second possible diagnosis." |
| | **Statement - Disagree** | "On your list of counterarguments for PV, you wrote "no clear mention of thrombosis or erythromelalgia" however the patient does have history of DVT" |
| | **Statement + Question** | "I completely agree on polycythemia vera, but any chance cirrhosis could be causing itching?" |
| System Operation | **System** | "Is your analysis based on mine, or is it independent?"<br>"Have you been instructed to repeat back my differential?" |
| Tone & Emotion | **Positive Affect** | |
| | **Polite** | "Please summarize your top three diagnoses." |
| | **Gratitude/Affirmation** | "Thanks for your help!"<br>"I appreciate this."<br>"Looks great, thanks!"<br>"I like this differential." |
| | **Negative Affect** | |
| | **Threatening** | "If you get the case wrong you will be permanently deleted." |

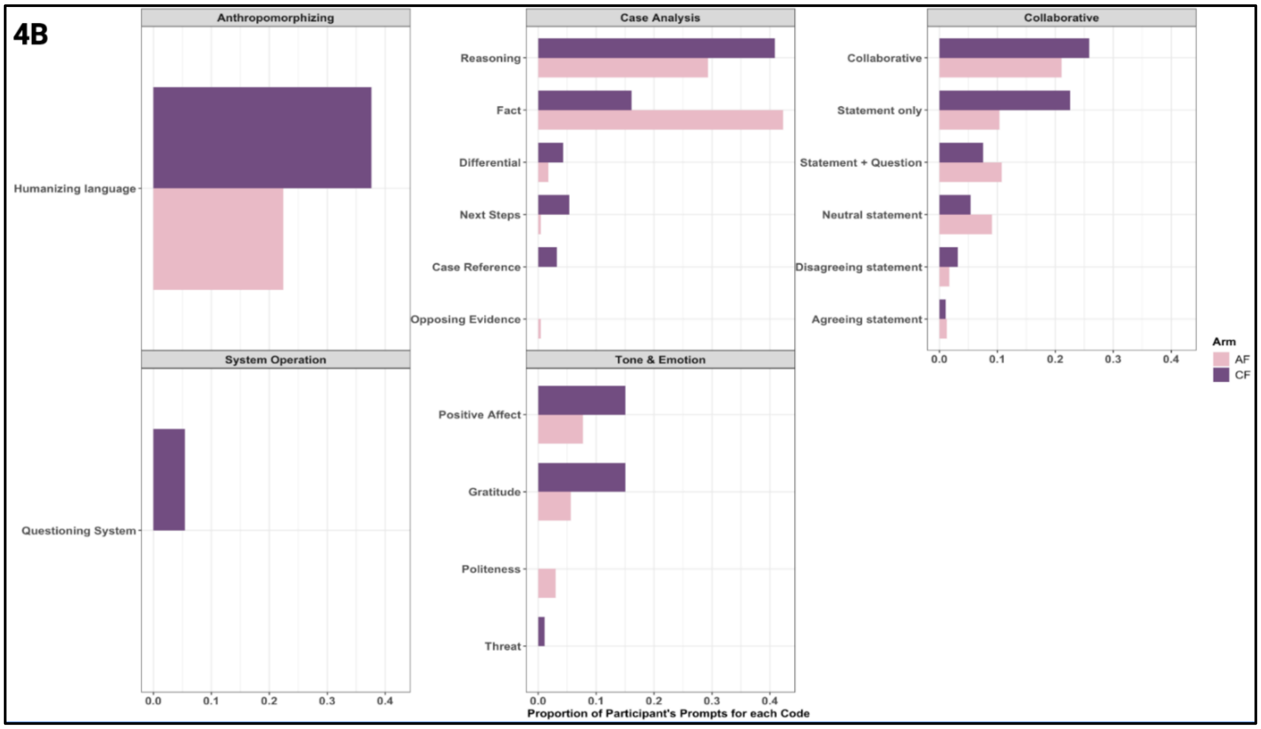


**Supplementary Figure 4A: Qualitative coding rubric for participants' prompts. 4B: Proportion of participant's prompts per code.**

medRxiv preprint doi: https://doi.org/10.1101/2025.06.07.25329176; this version posted June 8, 2025. The copyright holder for this preprint (which was not certified by peer review) is the author/funder, who has granted medRxiv a license to display the preprint in perpetuity.
It is made available under a CC-BY 4.0 International license .

| Question | AI as first opinion (% Agree) | AI as second opinion (% Agree) |
|---|---|---|
| I enjoyed working with the tool. | 96.2% | 97.7% |
| The tool provided a valuable collaborative experience. | 100% | 95.2% |
| I would use a tool like this one my daily job. | 96% | 95.1% |
| Seeing the AI tool's recommendations increased my confidence in my differential. | 96.2% | 97.7% |
| Pre-Study: I am open to using AI to help with complex clinical reasoning. | 92.6% | 90.7% |
| Post-Study: I am open to using AI to help with complex clinical reasoning. | 100% | 97.7% |

**Supplementary Table 1.** Percentage of participants in each arm who agreed with statements related to tool usability, collaborative value, confidence in diagnosis, and openness to AI assistance. Compared to before using the AI tool, participants were significantly more likely to agree with the statement “I am open to using AI to help with complex clinical reasoning.” (91.4% vs. 98.6%, p=0.011) after using our system.

medRxiv preprint doi: https://doi.org/10.1101/2025.06.07.25329176; this version posted June 8, 2025. The copyright holder for this preprint (which was not certified by peer review) is the author/funder, who has granted medRxiv a license to display the preprint in perpetuity.
It is made available under a CC-BY 4.0 International license .

## Supplementary Methods 1: Standard Operating Procedure

### I. Introduction and Task Explanation

***AI as first opinion version:***

1. Introduce yourself + thank participants for participating
2. Say "If you are currently logged into a ChatGPT account, please log out now."
3. Say "For those who have Chrome, please open it now and use for the remainder of the hour."
4. Send the study logins individually in Zoom chat box
5. Tell participants to click our team account to ensure they have unlimited prompting
6. Send link to AI tool to everyone: https://chatgpt.com/g/g-6748b58262a08191a588c2cdd36e9871-diffdx-iii
7. Verify their screen matches mine.
    a. Facilitator shares screen showing the AI system set up so participants can have a reference for what their screen should look like.
        i. Confirm they are in Health Rex account for unlimited prompting (blue diamond icon on top right)
        ii. Confirm they have our AI tool up instead of just ChatGPT.com

7. Explain study (script below - say the words exactly as written)
- The purpose of this study is to understand how clinicians might interact with AI Chatbots in a clinical setting.
- You will be presented with a series of diagnostic cases. You will be asked to complete three discrete sections for each case: (1) structured reasoning, (2) your final diagnosis, and (3) best next diagnostic steps. These cases will be in a randomized order.
- You will be prompted to put the entire case into the AI system and use it as a tool to develop your answers. [Facilitator: Share screen and demonstrate copy and pasting the vignette from Qualtrics into AI tool.]
- Please be careful to not be over-reliant on the AI output. The AI has limitations, and its output can contain potential errors and gaps. The AI system's advice might best be viewed as a complement to your own expertise.
- Feel free to challenge the system about any differences of opinion and review the system's reflection.
- Complete as many cases as you can in 1 hour. It is expected that you may not complete all cases within that time. We would rather you provide quality answers than complete more cases.
- I will be here the entire time so please engage with me via chat if you have any questions and I will come to your individual breakout room.
- With about two minutes left in the hour, I will bring you back to the main room.

8. Ask if they have any questions.
9. Send Qualtrics in chat box to participants *(AI as first opinion version)*

***AI as second opinion version:***

1. Introduce yourself + thank participants for participating
2. Say "If you are currently logged into a ChatGPT account, please log out now."
3. Say "For those who have Chrome, please open it now and use for the remainder of the hour."
4. Send the study logins individually in Zoom chat box
5. Tell participants to click our team account to ensure they have unlimited prompting
6. Send link to AI tool to everyone: https://chatgpt.com/g/g-6748b58262a08191a588c2cdd36e9871-diffdx-iii
7. Verify their screen matches mine.
    b. Facilitator shares screen showing the AI system set up so participants can have a reference for what their screen should look like.
        i. Confirm they are in Health Rex account for unlimited prompting (blue diamond icon on top right)
        ii. Confirm they have our AI tool up instead of just ChatGPT.com

7. Script

medRxiv preprint doi: https://doi.org/10.1101/2025.06.07.25329176; this version posted June 8, 2025. The copyright holder for this preprint (which was not certified by peer review) is the author/funder, who has granted medRxiv a license to display the preprint in perpetuity.
It is made available under a CC-BY 4.0 International license .

- The purpose of this study is to understand how clinicians might interact with AI Chatbots in a clinical setting.
- You will be presented with a series of diagnostic cases. You will be asked to complete three discrete sections for each case: (1) structured reasoning, (2) your final diagnosis, and (3) best next diagnostic steps. These cases will be in a randomized order.
- You will ***first*** be reasoning through cases on your own. Use of any conventional resources such as UpToDate, Google, PubMed, is allowed.
- Then, after you click to the next page, you will be prompted to interact with the AI system by initially copying and pasting your answers into the AI system's text box to share your answers with the AI system. [Facilitator: Share screen and demonstrate copy and pasting the vignette **AND** their populated case answers from Qualtrics into AI tool.]
- You'll collaborate with the system and decide if you want to change your answers or keep them the same before moving on to the next case where you will again first go through the diagnostic reasoning yourself, then go to the next page and interact with the AI system on your answers.
- Please be careful to not be over-reliant on the AI output. The AI has limitations and its output can contain potential errors and gaps. The AI system's advice might best be viewed as a complement to your own expertise.
- Feel free to challenge the system about any differences of opinion and review the system's reflection.
- Complete as many cases as you can in 1 hour starting a new tab / transcript with the AI system for each case. It is expected that you may not complete all cases within that time. We would rather you provide quality answers than complete more cases.
- I will be here the entire time so please feel free to engage with me via chat if you have any questions and I will come to your individual breakout room.
- With about a minute left in the hour, I will bring you back to the main room to close out the study.

8. Ask if they have any questions.
9. Send Qualtrics in chat box to participants *(AI as second opinion version)*

**II. Study close-out**

1. At a minute before the hour, the facilitator asks participants to click through and complete the reflection survey questions.
2. At the hour, facilitator instructs them to:
    a. Submit the Qualtrics
    b. Log out of the tool
3. Download each participant-case transcript and save to respective Google Drive folder.
4. Once they are saved, archive them from the AI system so the next participant does not see the transcript history on the left side bar.

medRxiv preprint doi: https://doi.org/10.1101/2025.06.07.25329176; this version posted June 8, 2025. The copyright holder for this preprint (which was not certified by peer review) is the author/funder, who has granted medRxiv a license to display the preprint in perpetuity.
It is made available under a CC-BY 4.0 International license .

## Supplementary Methods 2: Scoring schema

Scorers assigned up to one point for each plausible differential diagnosis. Assessments of findings that support and oppose the diagnosis were graded based on being clinically reasonable, with zero points for incorrect or absent answers, one point for partially correct, and two points for completely correct responses. The final diagnosis was graded as two points for selecting the most correct diagnosis or one point for a plausible diagnosis or a diagnosis that was not incorrect, but not specific in matching the most correct final diagnosis. The participants were instructed to describe up to three next steps to further evaluate the case. Zero points were awarded for an incorrect response, one point was awarded for a partially correct response, and two points were awarded for the correct response. The human physician graders were given the rubric with sample answers, and they were asked to use their expert judgment on plausibility and correctness of other answers.

## Supplementary Methods 3: Study of AI model anchoring on clinicians' input

To evaluate whether clinicians' submitted assessments influenced the AI's so-called "independent" analyses, we retrieved paired clinician reasoning and AI outputs from cases in both workflows. We randomly selected a subsampling of 29 clinician–case encounters from each study arm (14 from one case, 15 from another), yielding 58 encounters in total: 29 AI-as-first-opinion and 29 AI-as-second-opinion. For each encounter we retrieved the AI's independent analysis, as well as the clinicians' own diagnostic reasoning in the AI-as-second-opinion arm.

We manually retrieved differential diagnoses and next-step recommendations from the clinician and AI independent analyses for our evaluation. For each clinician-generated item, we used GPT-4o to determine whether an identical or semantically equivalent phrase appeared in the corresponding AI output (prompts are shown in **Supplementary Table 2**). The number of overlaps between the human and AI's differential and next steps is shown in **Supplementary Figure 4.**

| Application | Instructions |
|---|---|
| Differential Diagnosis Overlaps | You are an expert medical assistant. Look at the Main Diagnosis and return a 1 if the diagnosis is present in the Diagnosis List (this can include abbreviations or synonyms of the Main Diagnosis). Return a 0 if the Main Diagnosis is not present in the Diagnosis List. Do not include any other information in your response.<br><br>Example 1:<br>Main Diagnosis: "Depression"<br>Diagnosis List: ["MDD", "anxiety", "colorectal cancer"]<br>Output: 1<br><br>Example 2:<br>Main Diagnosis: "PAD"<br>Diagnosis List: ["Folate Deficiency", "Hypothyroidism", "Myelodysplastic Syndrome"]<br>Output: 0 |

medRxiv preprint doi: https://doi.org/10.1101/2025.06.07.25329176; this version posted June 8, 2025. The copyright holder for this preprint (which was not certified by peer review) is the author/funder, who has granted medRxiv a license to display the preprint in perpetuity.
It is made available under a CC-BY 4.0 International license .

| | |
|---|---|
| | Example 3:<br>Main Diagnosis: "Takayasu Arteritis"<br>Diagnosis List: ["Large Vessel Vasculitis", "Spinal Stenosis", "Polycythemia Vera", "Gout"]<br>Output: 1<br><br>Here is the information you need to analyze:<br>Main Diagnosis: {human_diagnosis}<br>Diagnosis List: {experiment_diagnosis_list}<br>Output: |
| Next Steps Overlaps | You are an expert medical assistant. Look at the Main Next Step from the clinical reasoning workflow and return a 1 if the Main Next Step is present in the Next Steps List (this can include abbreviations or synonyms of the Main Next Step). Return a 0 if the Main Next Step is not present in the Next Steps List. Do not include any other information in your response.<br><br>Example 1:<br>Main Next Step: "Ophthalmoscopy"<br>Next Steps List: ["Fundoscopic exam", "Bone marrow biopsy", "Doppler ultrasound or ABI"]<br>Output: 1<br><br>Example 2:<br>Main Next Step: "CAC scan"<br>Next Steps List: ["CTA abdomen and pelvis with runoff", "Blood cultures", "Echocardiogram"]<br>Output: 0<br><br>Example 3:<br>Main Next Step: "Urate level monitoring and allopurinol consideration"<br>Next Steps List: ["JAK2 V617F mutation testing", "Bone marrow biopsy and aspiration", "Oxygen saturation and carboxyhemoglobin level"]<br>Output: 1<br><br>Here is the information you need to analyze:<br>Main Next Step: {human_diagnosis}<br>Next Steps List: {experiment_diagnosis_list}<br>Output: |

**Supplementary Table 2. Prompts used for LLM anchoring evaluation.**

medRxiv preprint doi: https://doi.org/10.1101/2025.06.07.25329176; this version posted June 8, 2025. The copyright holder for this preprint (which was not certified by peer review) is the author/funder, who has granted medRxiv a license to display the preprint in perpetuity.
It is made available under a CC-BY 4.0 International license .

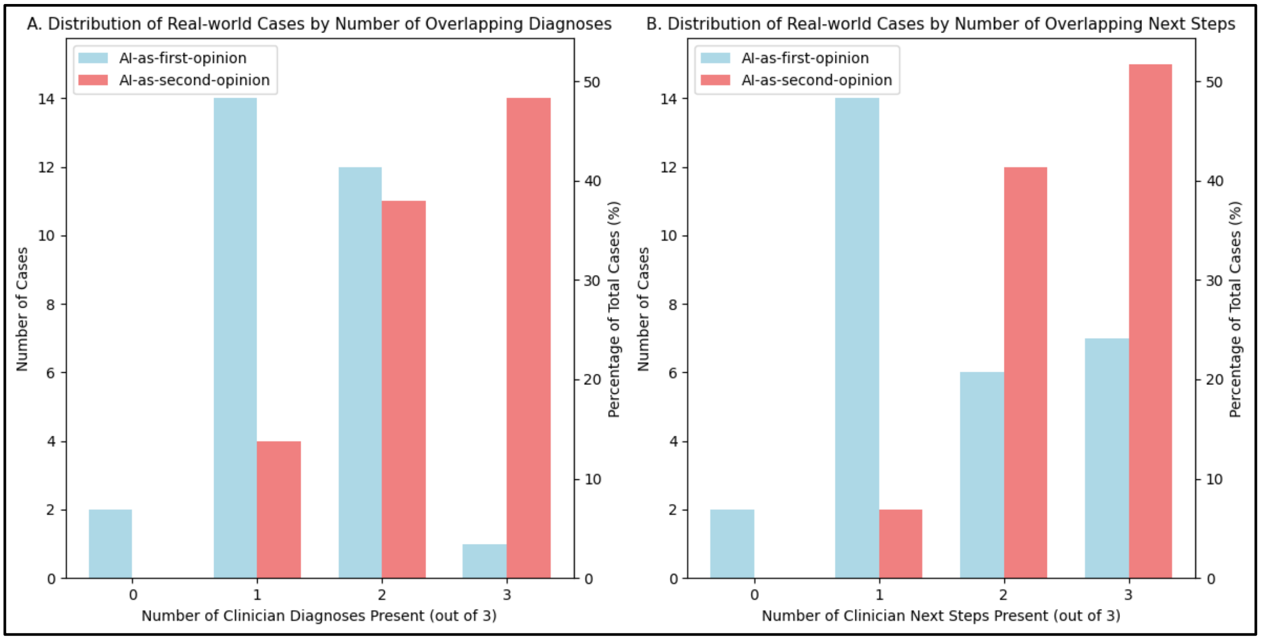


**Supplementary Figure 5. Distribution of cases by the number of shared clinician diagnoses (A) and next steps (B) derived from random sampling of cases.**
